# *MECP2* Mutation Interrupts Nucleolin–mTOR–P70S6K Signaling in Rett Syndrome Patients

**DOI:** 10.3389/fgene.2018.00635

**Published:** 2018-12-19

**Authors:** Carl O. Olson, Shervin Pejhan, Daniel Kroft, Kimia Sheikholeslami, David Fuss, Marjorie Buist, Annan Ali Sher, Marc R. Del Bigio, Yehezkel Sztainberg, Victoria Mok Siu, Lee Cyn Ang, Marianne Sabourin-Felix, Tom Moss, Mojgan Rastegar

**Affiliations:** ^1^Regenerative Medicine Program, and Department of Biochemistry and Medical Genetics, Max Rady College of Medicine, Rady Faculty of Health Sciences, University of Manitoba, Winnipeg, MB, Canada; ^2^Faculty of Medicine, University of Toronto, Toronto, ON, Canada; ^3^Department of Pathology, Max Rady College of Medicine, Rady Faculty of Health Sciences, University of Manitoba, Winnipeg, MB, Canada; ^4^Department of Molecular and Human Genetics, Baylor College of Medicine, Houston, TX, United States; ^5^Division of Medical Genetics, Department of Paediatrics, Schulich School of Medicine, Western University, London, ON, Canada; ^6^Department of Pathology, Schulich School of Medicine and Dentistry, Western University, London, ON, Canada; ^7^Cancer Division of the Quebec University Hospital Research Centre, Department of Molecular Biology, Medical Biochemistry and Pathology, Faculty of Medicine, Laval University, Quebec City, QC, Canada

**Keywords:** *MECP2* mutations, Rett syndrome, human brain tissues, DNA methylation, ribosome biogenesis, mTOR, nucleolin, protein translation

## Abstract

Rett syndrome (RTT) is a severe and rare neurological disorder that is caused by mutations in the X-linked *MECP2* (methyl CpG-binding protein 2) gene. MeCP2 protein is an important epigenetic factor in the brain and in neurons. In *Mecp2*-deficient neurons, nucleoli structures are compromised. Nucleoli are sites of active ribosomal RNA (*rRNA*) transcription and maturation, a process mainly controlled by nucleolin and mechanistic target of rapamycin (mTOR)–P70S6K signaling. Currently, it is unclear how nucleolin–*rRNA*–mTOR–P70S6K signaling from RTT cellular model systems translates into human RTT brain. Here, we studied the components of nucleolin–*rRNA*–mTOR–P70S6K signaling in the brain of RTT patients with common T158M and R255X mutations. Immunohistochemical examination of T158M brain showed disturbed nucleolin subcellular localization, which was absent in *Mecp2*-deficient homozygous male or heterozygote female mice, compared to wild type (WT). We confirmed by Western blot analysis that nucleolin protein levels are altered in RTT brain, but not in *Mecp2*-deficient mice. Further, we studied the expression of *rRNA* transcripts in *Mecp2*-deficient mice and RTT patients, as downstream molecules that are controlled by nucleolin. By data mining of published ChIP-seq studies, we showed MeCP2-binding at the multi-copy *rRNA* genes in the mouse brain, suggesting that *rRNA* might be a direct MeCP2 target gene. Additionally, we observed compromised mTOR–P70S6K signaling in the human RTT brain, a molecular pathway that is upstream of *rRNA*–nucleolin molecular conduits. RTT patients showed significantly higher phosphorylation of active mTORC1 or mTORC2 complexes compared to age- and sex-matched controls. Correlational analysis of mTORC1/2–P70S6K signaling pathway identified multiple points of deviation from the control tissues that may result in abnormal ribosome biogenesis in RTT brain. To our knowledge, this is the first report of deregulated nucleolin–*rRNA*–mTOR–P70S6K signaling in the human RTT brain. Our results provide important insight toward understanding the molecular properties of human RTT brain.

## Introduction

Methyl CpG-binding protein 2 gene was discovered in 1992, encoding for MeCP2 as an important member of the DNA methyl binding proteins (MBP) (Lewis et al., 1992). MeCP2 is an epigenetic regulator with crucial functions in the brain and in neurons (Delcuve et al., 2009; Ezeonwuka and Rastegar, 2014; Liyanage et al., 2014). *De novo* mutations of the X-linked *MECP2* gene are the underlying cause of ∼95% cases of RTT (Amir et al., 1999). RTT is a severe and rare progressive neurodevelopmental disease in females (1:10,000), with few cases of reported male patients (Liyanage and Rastegar, 2014). RTT patients appear normal at the beginning of their life, but by 6–18 months, they exhibit developmental regression and loss of acquired skills, along with neurological symptoms that may include seizures, ataxia, and autistic characteristics.

It is well established that *MECP2* deficiency in neurons is associated with compromised protein synthesis (Li et al., 2013), a fundamental process in all cells including neurons. Protein synthesis is tightly regulated and has multiple rate-limiting steps. Of those steps, ribosome biogenesis and *rRNA* synthesis are largely controlled (Moss and Langlois, 2007). Eukaryotic ribosomes are subcellular organelles made of *rRNA* transcripts and a multitude of ribosomal proteins. The process of *rRNA* synthesis, in turn, is a rate-limiting step for ribosome biogenesis. The multi-copy *rRNA* genes are initially transcribed by polymerase I as *45S pre-rRNA* precursors in the nucleolus that are processed into *18S*, *28S*, and *5.8S rRNA*s (Moss, 2004; Moss and Langlois, 2007). RNA polymerase I activity is controlled by nucleolin and the mTOR-P70S6K ribosomal protein pathway. It has been reported that the *28S* and *18S rRNA* transcripts are reduced in murine *Mecp2*-deficient neurons (Gabel et al., 2015), and that the mTOR signaling is impaired in RTT mouse models (Ricciardi et al., 2011). However, no study has been done in the human RTT brain, and there is no report if the nucleolin levels are interrupted in human RTT brain. As nucleolin controls *rRNA* synthesis/ribosome biogenesis, and this process is also controlled by mTOR–P70S6K signaling, we hypothesized that MeCP2 mutations in human RTT brain would be associated with deregulation of nucleolin, *rRNA* transcripts, and mTOR–P70S6K signaling. Previous reports have highlighted a role for MeCP2 in organizing neuronal nucleoli structure during embryonic development (Singleton et al., 2011), while pointing toward MeCP2 recruitment at the nucleolar periphery of Purkinje cells in mice cerebellum. This is suggestive of MeCP2 binding to methylated *rRNA* genes at peri-nucleolar parts of the nucleus (Payen et al., 1998), introducing *rRNA* genes as potential direct target genes of MeCP2. While these studies highlight a functional importance for MeCP2 in embryonic neuronal nucleoli and Purkinje cells of mice, it is unclear if human RTT cerebellum has nucleolar deficits. MeCP2 levels are highest in neurons, and of different brain regions, cerebellum has the highest neuronal density (Marzban et al., 2014). The cerebellum has established links with autism, cognitive characteristics, ataxia, and memory function (some of the main RTT phenotypic characteristics), and has been studied for RTT-associated research on the mechanism of disease (Ben-Shachar et al., 2009; Rangasamy et al., 2016; Rastegar, 2017). Therefore, in our studies we focused on human RTT cerebellum.

Here, we report that human RTT brain shows deregulation of multiple molecules upstream of protein translation, altered nucleolin protein levels, and mTOR–P70S6K pathway. To our knowledge, the possible link between MeCP2, nucleolin levels, and mTOR–P70S6K pathway in RTT and other MeCP2-associated neurological disorders (i.e., MDS) is a novel concept that is being reported. Data presented in this study suggest a potential regulatory role for MeCP2 that may lead to a better understanding of MeCP2-associated disease pathobiology.

## Materials and Methods

### Immunohistochemistry

Dissected mouse brain fixation in ice-cold freshly de-polymerized 2% PFA (0.16 M sodium phosphate buffer, pH 7.4 with PFA) was followed by incubation in cryoprotectant (25 mM sodium phosphate buffer, pH 7.4, 10% sucrose, and 0.04% NaN_3_) at 4°C for at least 24h. Ten micron mouse brain cryosections were processed on to gelatinized slides and stored at −20°C. Slides were air-dried at room temperature prior to use. Human brain sections (5 μm) were incubated in an oven at 60°C for 30 min, then deparaffinized using sequential incubations of 4 × 5 min xylene, 2 × 1 min 100% ethanol, 1 × 1 min 95% ethanol, 1 × 1 min 70% ethanol, 1 × 1 min running tap water, and 1 × 1 min distilled de-ionized water. De-paraffinized human brain sections were treated using Tris-EDTA antigen retrieval buffer (10 mM Trizma base, 1 mM EDTA, pH 9.0, 0.05% Tween-20) or citrate antigen retrieval buffer (10 mM sodium citrate, pH 6.0, 0.05% Tween-20) both at boiling temperature for 20 min, followed by 3 min air-cooling and 3 × 5 min TBS (50 mM Trizma base, pH 7.6, 1.5% NaCl) wash. Human and mouse brain sections were permeabilized for 20 min in TBS-Tr (50 mM Trizma base, pH 7.6, 1.5% NaCl, 0.3% Triton X-100) and pre-blocked with 10 or 20% normal goat serum (NGS) in TBS-Tr overnight at 4°C. Immunohistochemistry (IHC) was performed using rabbit polyclonal anti-nucleolin (Abcam, ab22758) primary antibody in TBS-Tr with serum. Secondary antibody, goat anti-rabbit Alexa 594 (Thermo Fisher, A11037), was also diluted in TBS-Tr with serum and applied for 1 h at room temperature followed by washes using 3 × 20 min TBS-Tr and 1 × 15 min Tris–HCl buffer (50 mM Trizma base, pH 7.4). Sudan Black counterstaining (0.1% w/v in 70% EtOH) for 30 min followed secondary antibody washes for citrate antigen retrieval samples, followed by 1 × 5 min wash with 70% EtOH and 3 × 5 min wash with TBS. DAPI counterstaining and washes with Tris–HCl was performed followed by application of Prolong Gold (Thermo Fisher, P36930) antifade and cover slipping. Immunolabeling was detected using an Axio Observer Z1 inverted microscope and LSM710 confocal microscope (Carl Zeiss Canada Ltd.), as previously described (Olson et al., 2014). Images were obtained and analyzed using Zen (Carl Zeiss Canada Ltd.) software, and assembled into figures using Adobe Photoshop C5 and Adobe Illustrator C5. Please refer to Supplementary Tables S1, S2 for the list of primary and secondary antibodies.

### Western Blot

Nuclear and cytoplasmic extraction from brain tissues were carried out using NE-PER^TM^ Nuclear and Cytoplasmic Extraction Kit (Thermo Scientific Inc., 78835) as per the manufacturer’s instructions, and as we reported (Olson et al., 2014). Total protein cell extracts were done by high salt protocol as we have reported (Lahuna et al., 2000; Rastegar et al., 2000; Wu et al., 2001; Nolte et al., 2006). WB experiments and quantification of the signals was performed as we reported (Olson et al., 2014; Nagakannan et al., 2016). AlphaEaseFC (version 6.0.0, Alpha Innotech) software was used for quantification. Please refer to Supplementary Tables S1, S2 for the list of primary and secondary antibodies.

As loading control for WBs with total cell extracts, we used GAPDH as a commonly used housekeeping protein, which appeared to be consistently detectable across different samples, when the same amount of protein was loaded for each sample. Similarly, GAPDH signals in the cytoplasmic extracts remained constant, providing a reliable indication that comparable level of protein samples are loaded for each sample. This is in agreement with GADH being reported as a key enzyme for glycolysis (Bruns and Gerald, 1976; Sirover, 1999) in the cytoplasm. We also used GAPDH as a loading control for nuclear extracts with consistent detection among different samples when the same amount of nuclear protein extracts were loaded onto the gels. This is in accordance with the reported role of nuclear GAPDH in maintenance and protection of telomeric DNA (Sundararaj et al., 2004), and also regarding its functional role in controlling histone H2B expression (Zheng et al., 2003; Nicholls et al., 2012). While the level of loaded proteins in the cytoplasmic and nuclear extracts were verified by GAPDH signals, detection of histone H3 (pan H3 and acetylated H3) and S100 protein was used to verify the quality of extracted nuclear and cytoplasmic extracts, respectively.

### Quantitative Real-Time PCR (qRT-PCR)

Total RNA from murine and human brain regions was extracted by Trizol, as we reported elsewhere (Rastegar et al., 2004; Kobrossy et al., 2006). Quantitative RT-PCR was done using SYBR Green-based RT2 qPCR Master Mix (Applied Biosystems, 4367659) in an Applied Biosystems Fast 7500 Real-Time PCR machine. The threshold cycle value (Ct) for each gene was obtained from the Applied Biosystems Fast 7500 Real-Time PCR machine and the values were normalized against a housekeeping gene (*Gapdh)*. This was followed by obtaining the ΔCt values for each one of the samples, by calculating the relative levels of each gene by calculating 2^−ΔCt^ for each sample. Analysis was done by Microsoft Excel 2010 and 2^−ΔCt^ values of each gene that were transferred to GraphPad Prism 6.0, for generating the final graphs, a similar analysis that we reported previously (Liyanage et al., 2013, 2015). Statistical significance was determined by Welch’s *t*-test, with ^∗∗∗∗^*p* < 0.0001, ^∗∗∗^*p* < 0.001, ^∗∗^*p* < 0.01, or ^∗^*p* < 0.05. The sequence of the primers used in RT-qPCR reactions are as following: mouse *nucleolin*: forward: 5′-AAGCAGCACCTGGAAAACG-3′, reverse: 5′-TCTGAGCCTTCTACTTTCTGTTTCTTG-3′ (Monte et al., 2013); mouse *GAPDH*: forward: 5′-ATGTCGTGGAGTCTACTGG-3′, reverse: 5′-GTGGTGCAGGATGCATTGC-3′; mouse *45s rRNA*: forward: 5′-GAGAGTCCCGAGTACTTCAC-3′, reverse: 5′-GGAGAAACAAGCGAGATAGG-3′ (Chen et al., 2008); human/mouse *28s rRNA*: forward: 5′-AGAGGTAAACGGGTGGGGTC-3′, reverse: 5′-GGGGTCGGGAGGAACGG-3′ (Uemura et al., 2012); human/mouse *18s rRNA*: forward: 5′-GATGGTAGTCGCCGTGCC-3′ (Uemura et al., 2012); reverse: 5′-GCCTGCTGCCTTCCTTGG-3′; human *GAPDH*: forward: 5′-CCACTCCTCCACCTTTGAC-3′, reverse: 5′-ACCCTGTTGCTGTAGCCA-3′; human *nucleolin*: forward: 5′-AGCAAAGAAGGTGGTCGTTT -3′, reverse: 5′-CTTGCCAGGTGTGGTAACTG -3′; human *45S rRNA*: forward: 5′-CTCCGTTATGGTAGCGCTGC-3′, reverse: 5′-GCGGAACCCTCGCTTCTC-3′.

### Correlation Analysis and Ratios

The correlations between protein contents of mTOR, mTORC1 (2448), mTORC2 (2481), P70S6K, and phosphorylated P70S6K were determined by Pearson’s correlation analysis. WB signals were normalized against GAPDH loading control, and individual values for each signal were compared to the average of the controls in that blot; this was done in order to render values from different technical replicates comparable. We then calculated Pearson’s correlation coefficient (*r*) for the normalized values of each pair of molecules within the RTT patients and controls. The power of correlation is reported as follows: very weak/poor, 0 < *r* < 0.3; moderate/medium, 0.3 < *r* < 0.4; strong, 0.4 < *r* < 0.7; and very strong, 0.7 < *r* < 1.0. Due to the nature of the data, significance was not computed.

Ratios between molecules were computed for control tissues (*n* = 3), average RTT patients (*n* = 4), and each RTT patient individually. The average of normalized WB signals for each sample was compared between the phosphorylated proteins to the corresponding total protein to obtain ratios. Error bars for each sample represent the SEM for the RTT and control ratios. Significance was computed using FDR-adjusted multiple *t*-tests, with an alpha of 0.05.

### Sequence Data Mining

The raw sequence data was obtained for both ChIP and input DNA samples (GSM1464563 and GSM1464564) (Gabel et al., 2015) and was then aligned to the mouse genome version MmGRCm38 to which a single copy of the mouse *rDNA* repeat sequence (GenBank BK000964v3), which was added as an additional chromosome by using Bowtie2 (Langmead and Salzberg, 2012). For practicality, the source of the *rRNA* repeat was placed at the EcoRI site at 30,493 in a way that the *pre-rRNA* initiation site now is located at the nucleotide 14,815.

The deconvoNorm.py script was then used to normalize the data^1^ (Mars et al., 2018). Briefly, the aligned reads were extended to 100 bp and the coverage was calculated by using BEDtools (Quinlan Lab, University of Utah). The resultant data was then converted to the RPM and the sample DNA coverage was normalized to the input DNA coverage (sample/input) for each of the base positions. The resulting normalized BED files were then converted to BEDgraph format and were visualized using IGV (IGV 2.3, Broad Institute). WGBS data (GSM1173783) (Lister et al., 2013) was also analyzed for the methyl-dC by alignments to the same composite mouse genome by using the Bismark v0.10 (Krueger and Andrews, 2011), and Bowtie2, and were again visualized using IGV (IGV 2.3, Broad Institute).

### Ethical Approval and Consent to Participate

All experiments with mice were conducted according to the standards of the Canadian Council on Animal Care with the approval of the Office of Research Ethics of the University of Manitoba, in accordance with approved guidelines on animal experimentation. MeCP2 knockout transgenic mice *Mecp2*^*tm*1.1*Birdy*/−^ (null), heterozygote female (*Mecp2*^*tm*1.1*Bird* +/−^), and their WT counterparts were purchased from The Jackson laboratories, United States. Mice tissue harvest and outlined experimental procedures were peer-reviewed and approved under the “animal protocol number 16-031/1/2(AC-11190)” by the University of Manitoba Bannatyne Campus Protocol Management and Review Committee. Samples from *MECP2-Tg1* and *Tg3* mice were received from Dr. Huda Zoghbi, Baylor College of Medicine, Houston, TX, United States, as they previously reported (Samaco et al., 2012). The human tissue research has been reviewed and approved by the University of Manitoba Bannatyne Campus research ethics board and (Health Research Board protocol # HS20095 H2016:337). For donated T158M human RTT brain tissues, we obtained appropriate family consent to participate in research (through Dr. Victoria Siu, co-author), and the T158M RTT post-mortem brain tissues were collected from a 13-year-old female with RTT diagnosis. Control fixed cerebellum tissues are from age-matched female. Human brain tissues for RNA and protein extractions from RTT patients (R255X: c.763C > T nonsense mutation, 17 and 20 years old, case numbers #4516 and #4882; and G451T case number #4852) and control age-matched female tissues (17, 19, and 20 years old, case numbers #5446, #1347, and #5646) were received from NIH NeuroBiobank at the University of Maryland Brain and Tissue Bank, as frozen and formalin-fixed paraffin-embedded tissues.

### Clinical Information and History of the Rett Syndrome Patients

The T158M patient was born at 40 weeks gestation following an uncomplicated pregnancy, weighing 2984 g (25th percentile). She was the only child born to a 21-year-old mother and a 33-year-old father, and was diagnosed with RTT at the age of 2½ years. Her early developmental milestones were normal. At 6 months of age, she started grinding her teeth, rolled at 5 months, and crawled and took her first steps at 1 year. At 18 months, she began to regress, losing purposeful hand movements, the ability to walk and to speak, as well as developing severe constipation. Her eye contact became very poor, but was regained by 2½ years of age. From age 2, she was constantly mouthing or wringing her hands. She also exhibited hyperventilation and abdominal bloating. She had intermittent strabismus, and abnormal EEG with generalized grade 4 dysrhythmia. At 2 years, she began exhibiting repetitive hand movement characteristic of RTT, leading to her diagnosis. Genetic testing revealed a T158M mutation in the *MECP2* gene, confirming the RTT diagnosis. She was always a happy and passive child who never experienced a period of irritability in association with her regression. On examination at age 4, she showed constant handwringing and bruxism. Height was 93 cm (third percentile), weight 15.1 kg (25th percentile) and head circumference 48.2 cm (10th to 25th percentile). By age 8, she was having three types of seizures: staring spells with facial and arm twitching, tonic–clonic seizures, and apneic periods associated with cyanosis. At 9 years of age, she appeared to have multifocal myoclonus. Choking and aspiration episodes became frequent, necessitating recurrent admissions to the critical care unit for intubation and ventilation. By 10 years, she had developed a seizure disorder and respiratory dysfunction, showing characteristic autonomic fluctuations of heart rate and respiratory function. By age 12 years, her overall health and quality of life had decreased significantly to the point of respiratory insufficiency, requiring continuous BiPAP and frequent suctioning to maintain oxygenation and clear secretions. She died of respiratory failure after the age of 12. She passed away peacefully and her mother requested that her brain be donated for research into RTT. The post-mortem autopsy revealed a partially resolved subdural hemorrhage, mild enlargement of the lateral ventricles, and slight thinning of the posterior corpus callosum, with no other focal abnormalities noted. Upon analysis by microscopy, increased cell density and small pyramidal neurons were noted, as well as diffuse mild microglial activation in the white matter. No other abnormalities were noted.

The NIH case # 4516, R255X mutation, 20-year-old patient was a right-handed female, born vaginally at 43 weeks gestation. At around 16 months, development of her speech stagnated. Over the following 6 months, she began to lose motor function in her hands and bowels/bladder. At 2 years of age, she began to have focal onset unaware seizures lasting 5–8 s and occurring three times per week. She continued to deteriorate until her death at age 20. On autopsy, slight cerebral atrophy was noted. A post-mortem genetic analysis identified an R255X mutation in the *MECP2* gene.

The NIH case # 4882, R255X mutation, 17-year-old patient was born vaginally at 39.5 weeks gestation, after a prolonged rupture of membranes of 26 h. At birth, she was diagnosed to have torticollis, which was thought to be due to low intrauterine tone. This was corrected with special pillows. At 21 months of age, she was noted to have hypotonia and hyporeflexia, constant repetitive hand and foot movements, and brachycephaly. A genetic test at this time identified a C763T nonsense mutation in the *MECP2* gene translating into R255X mutation in the MeCP2 protein, confirming a diagnosis of RTT. The patient passed away at the age of 17 years old. On autopsy, no significant pathologic findings were identified.

The NIH case # 4852, G451T mutation, had a limited clinical history available. Her clinical course included kyphoscoliosis and epilepsy, which began in her early childhood. She died at the age of 19 years old and a neuropathological examination revealed a pale substantia nigra.

For further information, including PMI, and the storage years prior arriving to our lab for research, please refer to Supplementary Table S3.

**FIGURE 1:**
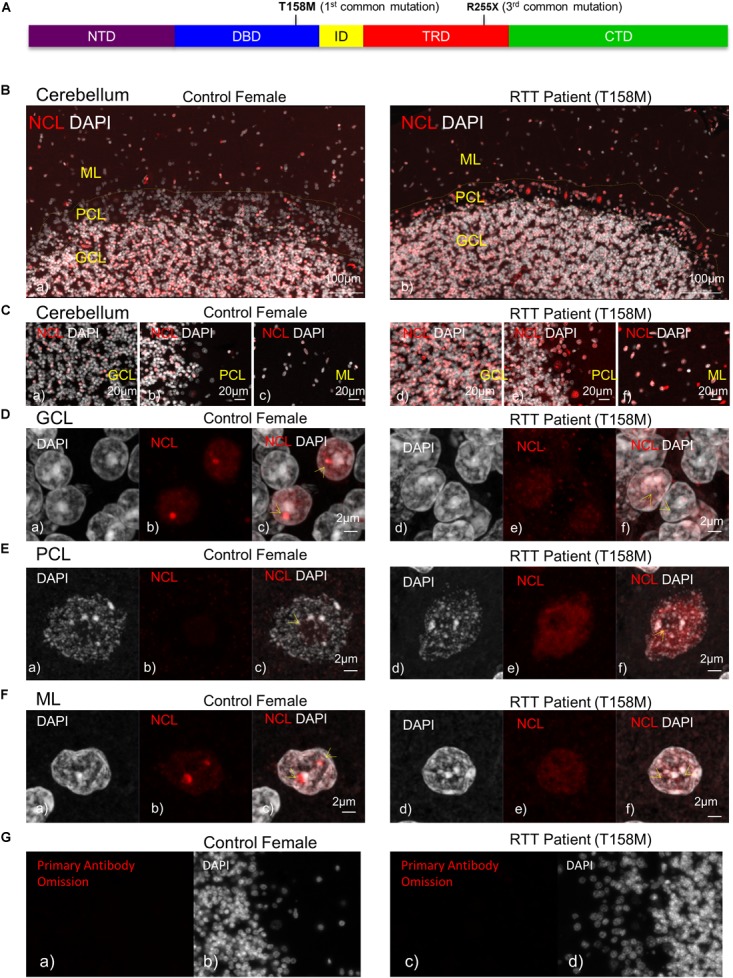
Detection of nucleolin protein in the cerebellum of T158M Rett syndrome (RTT) patient. **(A)** Schematic representation of MeCP2 protein. Position of T158M and R255X mutations within the MeCP2 functional domains is shown. **(B)** Microscopic image of post-mortem human cerebellum for nucleolin (red) and DAPI signals (white) in a female control and a T158M 13-year-old patient is shown, for the three cerebellum layers (granular, Purkinje, and molecular cell layers). **(C)** Higher magnification images for the three cerebellum layers in control **(a–c)** and T158M RTT patient **(d–f)** are shown. **(D–F)** Confocal images of nucleolin in the three layers of the human cerebellum are shown. Note that in all three layers, nucleolin signals are redistributed from the nucleolus into the nuclei. Scale bars represent 100 μm in **(B)**, 20 μm in **(C)**, and 2 μm in **(D–F)**. Yellow arrows point toward nucleoli structures. **(G)** Primary antibody omission in female control **(a,b)** and the T158M RTT patient **(c,d)** are shown. CTD, C-terminal domain; DBD, DNA-binding domain; GCL, granular cell layer; ID, intervening domain; ML, molecular layer; NCL, nucleolin; NTD, N-terminal domain; PCL, Purkinje cell layer; TRD, transcriptional repression domain.

## Results

### Nucleolin Protein Levels and Sub-Cellular Localization Are Deregulated in the Human T158M RTT Brain

**FIGURE 2:**
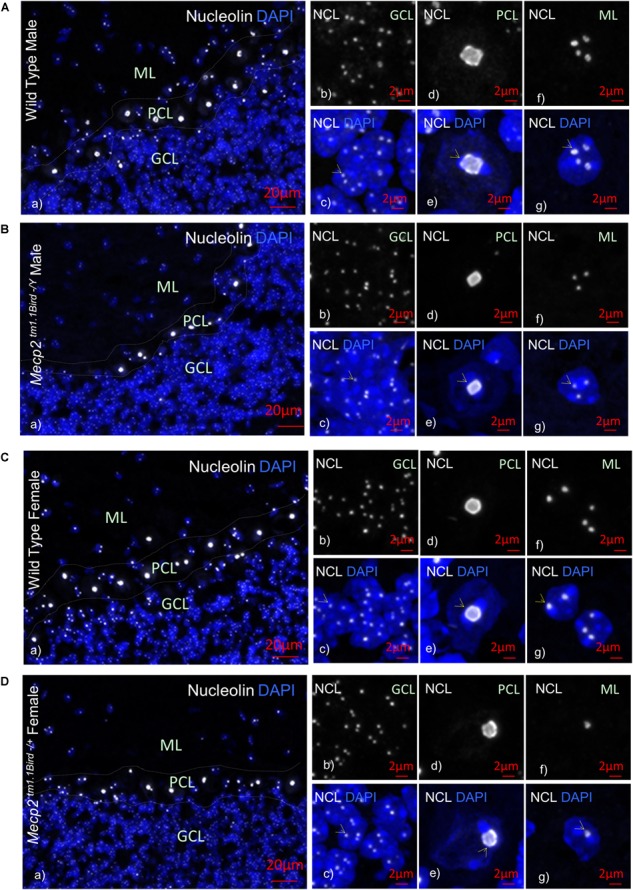
Nucleolin in the cerebellum of homozygous male *Mecp2*^*tm*1.1*BirdY*/−^, heterozygous female *Mecp2*^*tm*1.1*Bird*+/−^, and age-matched wild-type male/female mice. Immunohistochemical analyses are shown for cerebellum of wild-type male at approximately 6 weeks of age **(A)**, male null homozygous *Mecp2*^*tm*1.1*BirdY*/−^**(B)**, wild-type female **(C)**, and heterozygous female *Mecp2*^*tm*1.1*Bird*+/−^ **(D)**. Note that *Mecp2* is X-linked and heterozygote male or homozygote female does not exist in the *Mecp2*^*tm*1.1*Bird*^ mice. In **(A–D)**, panel **(a)** indicates an image of all cerebellum layers together to show nucleolin (white) and DAPI (blue); **(b,c)** granular cell layer (GCL) with **(b)** showing the nucleolin and **(c)** showing the overlay; **(d,e)** similar to **(b,c)** but for Purkinje cell layer (PCL); **(f,g)** similar to **(b,c)** and **(d,e)** but for molecular layer (ML). Scale bars represent 20 μm in **(Aa–Da)**, and 2 μm in all other images. Yellow arrows point toward nucleoli structures.

In order to study the impact of MeCP2 mutations in nucleoli structures in humans, we analyzed post-mortem cerebellum of a RTT patient with the most common MeCP2 mutation (T158M). This mutation is recognized as the highest frequency RTT-associated MeCP2 mutation and occurs in the MeCP2 DBD (Figure 1A). Nucleolin is a major nucleoli protein that makes up about 10% of total nucleolar proteins (Singleton et al., 2011; Tajrishi et al., 2011). We performed IHC analysis of post-mortem cerebellum tissues from the T158M 13-year-old RTT patient with a clinical RTT diagnosis (Figures 1B–F). We detected higher levels of nucleolin in the cerebellum ML, PCL, and GCL of the RTT patient compared to control tissue (Figures 1B–F). Especially in the Purkinje cells, a faint nucleolin staining was detected in DAPI-devoid regions of the nucleolus, but was detected at higher levels throughout the nucleus in the RTT patient (Figure 1E). Using confocal microscopy, we also detected a clear nucleolin staining in the nucleoli of the GCL and ML cells of the control cerebellum, but this appeared to be faint and distributed throughout the nucleus in the RTT T158M cerebellum (Figures 1D,F). No signal was detected in primary antibody omission control samples (Figure 1G). Comparative analysis of murine cerebellum in 6-week WT compared to null *Mecp2*^*tm*1.1*Birdy*/−^ brain tissues did not show significant differences in the nucleolar morphological organization analyzed by nucleolin staining (Figures 2A,B). Our observation in mice cerebellum was in agreement with a previous report of nucleolin staining in the cortex of adult *Mecp2*^*tm*1.1*Birdy*/−^ homozygous mice compared to WT male (Singleton et al., 2011). This is also in agreement with a previous report that detected compromised nucleolar structures in *Mecp2*-deficient neurons at the embryonic stage, which were corrected by adulthood in mice (Singleton et al., 2011). In murine cerebellum at 6 weeks of age, we did not detect any differences between the WT female and heterozygote *Mecp2*^*tm*1.1*Bird* −/+^ female (Figures 2A–D). Due to the X-linked nature of the *Mecp2/MECP2* gene, no homozygous female or heterozygote male mice are available for comparison studies. Comparing the results from T158M RTT patient and *Mecp2*-deficient transgenic mice, it is possible that there is differential regulation of nucleoli structures in mice and humans that might be detected in human MeCP2 mutant brain tissues. It is also possible that the effect of total MeCP2 protein loss (in *Mecp2*^*tm*1.1*Bird*^ mice) would be different from an RTT patient that has the full-length protein, but with a specific point mutation.

**FIGURE 3:**
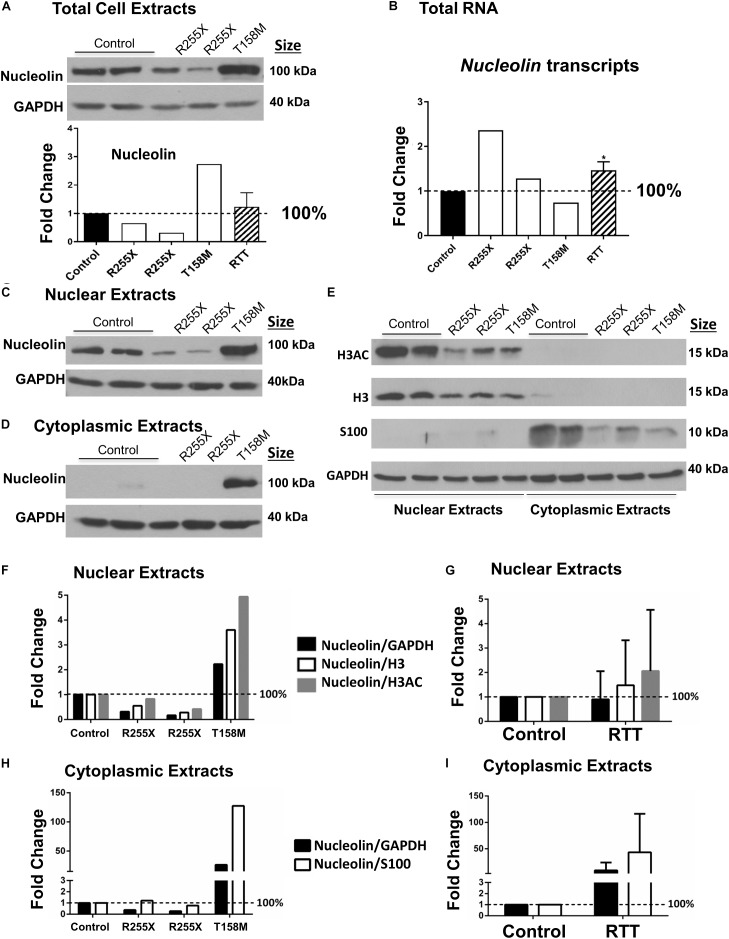
Nucleolin in the human Rett syndrome (RTT) cerebellum and controls. **(A)** Western blot (WB) analysis of total cell extracts of controls and RTT patients. The data are shown with the following samples in the order of controls (NIH NeuroBiobank case numbers #5646 and #5446) and RTT patients (R255X: c.763C>T nonsense mutation, 20 and 17 years old, case numbers #4516 and #4882), and T158M cerebellum (brain received as donation by family members with appropriate consent for research). Averaged data for the three patients is shown in the “RTT” column. **(B)** Transcript level of *Nucleolin* is shown for human cerebellum. Human control and RTT patients are the same as in **(A)**. **(C)** Same as **(A)**, but for nuclear proteins. **(D)** Same as in **(A,C)**, but for cytoplasmic extracts. **(E)** Validation of nuclear and cytoplasmic fractions using antibodies against histone H3, H3 di-acetylation at K9–K14 (H3AC) as nuclear proteins, and astrocytic protein S100 as a cytoplasmic protein. The order of samples is the same as in **(A,C,D)**. **(F)** Quantification of nuclear nucleolin signals from C against GAPDH (loading control), H3, or H3AC as other ubiquitous nuclear proteins. **(G)** Combined quantification of the three RTT patients from **(F)**. **(H)** Quantification of cytoplasmic nucleolin signals from **(D)** against GAPDH (loading control), or S100 as other cytoplasmic protein in the brain. **(I)** Combined quantification of the three RTT patients from **(H)**. *N* = 2 for controls and values represent single RTT patients in **(F,H)** (the two R255X patients, and T158M patient). In **(A,B,G,I)**, *N* = 2 for controls and *N* = 3 ± SEM for RTT patients. Statistical significance was determined by Welch’s *t*-test with ^∗^*p* < 0.05 and two-way ANOVA.

Next, we asked if the higher nucleolin levels shown by IHC in T158M cerebellum are also detectable by a more quantitative analysis. We isolated protein extracts of the cerebellum from the T158M patient and control tissues for WB. Nucleolin protein levels in T158M female cerebellum were observed to be at higher levels compared to control cerebellum tissues (Figure 3A). Examination of *Nucleolin* transcripts did not show a direct correlation with the protein levels, as transcripts were found to be at the lowest levels in the T158M patient (Figure 3B).

In general, the severity of the disease in RTT and the associated phenotypes may vary depending on the type of *MECP2* genetic mutation and the affected functional domain of the protein (Liyanage and Rastegar, 2014). While T158M is the highest frequency of MeCP2 mutations in RTT, R255X is the highest frequency of MeCP2 mutation in the TRD, constituting the third most common RTT-associated mutation (Figure 1A). In two different cases of R255X mutations in RTT patients, nucleolin levels appeared to be below the control levels (Figure 3A), suggesting that altered nucleolin levels might depend on the type of *MECP2* mutation. Transcript analysis of *Nucleolin* mRNA level in the two R255X patients showed slightly increased *Nucleolin* transcripts compared to controls (Figure 3B), suggesting regulation at the level of nucleolin translation or turnover.

Immunohistochemistry examination of the nucleolin in the cerebellum tissues of these R255X patients compared to age- and sex-matched controls suggested lower detection of nucleolin in these patients (Supplementary Figures S1A–D). However, the quality of the tissues for IHC examination was largely reduced due to the long-term storage of these brain tissues in formalin (NIH Neurobiobank #4516: over 11 years, and NIH Neurobiobank 4882: over 9 years). In both cases, some levels of noise background were detected in primary omission controls slides for the two RTT patients (Supplementary Figures S1Ce,De), indicating that there is some level of auto-fluorescence in these tissues when trying to visualize the low levels of nucleolin by microscopy. It is important to note that although IHC results may point toward antigen detection in individual cells, WB experiments are more reliable for quantitative expression level studies in between different samples.

Next, we studied nucleolin levels in the nuclear and cytoplasmic fractions of the cerebellum from all three patients, as we had noticed change in nucleolin sub-cellular localization in the T158M patient. The T158M cerebellum showed higher levels of nucleolin in both nuclear and cytoplasmic fractions, while the two R255X and control cerebellum cytoplasmic extracts showed negligible nucleolin levels (Figures 3C,D). In order to ensure the highly detected nucleolin protein in the cytoplasmic fraction of the T158M is not a simple technical error due to the contamination of nuclear protein in this patient, we verified the quality of our nuclear–cytoplasmic fractions. Analysis of histone H3 detection along with its specific acetylation modification (H3AC: histone H3 di-acetyl K9–K14) as nuclear-specific proteins indicated that there is no nuclear contamination in the T158M cytoplasmic fraction (Figure 3E) as no H3 or H3Ac was detected in the T158M sample. Accordingly, examination of a cytoplasmic protein (S100) confirmed negligible detection in the nuclear extracts, compared to the cytoplasmic extracts (Figure 3E). We used a housekeeping protein (GAPDH) loading control on these experiments (Figure 3E). Examination of the nuclear extracts from RTT cerebellum showed lower levels of H3 and H3AC in all three patients compared to controls. Accordingly, cytoplasmic extracts of the RTT patients showed lower levels of S100 expression compared to controls (Figure 3E and Supplementary Figure S3). As GAPDH levels remained relatively consistent among controls and RTT patients, it is possible that lower levels of H3, H3AC, and S100 in RTT patients may have biological relevance. Regardless, quantification of nucleolin signals in the R255X patients normalized to GAPDH, H3, or H3AC in the nuclear extracts showed a trend of decreased nucleolin levels, which was more drastic when it was normalized to GAPDH (Figure 3F). In the T158M patient, nucleoli level was between twofold and fivefold higher than the control levels depending normalization to GAPDH loading control or H3, and H3AC nuclear proteins. Combination of the values from the three RTT patients did not show significant change from the controls, due to the opposite alteration of nucleolin in R255X (decreased) versus T158M patient (increased) protein levels (Figure 3G). Accordingly, detection of cytoplasmic nucleolin levels in the T158M RTT patient compared to GAPDH loading control or S100 cytoplasmic marker showed an increase of over 100-fold (when compared to S100 levels) (Figure 3H), but was not significant in R255X and T158M patients, when all three patients were combined together (Figure 3I). Importantly, all three patients showed reduced levels of nuclear histones (H3 and H3AC), as well as S100 cytoplasmic protein that appeared to be statistically significant (Supplementary Figures S3A–D). However, understanding the biological relevance of these differences and possible pathological implications requires further investigations.

**FIGURE 4:**
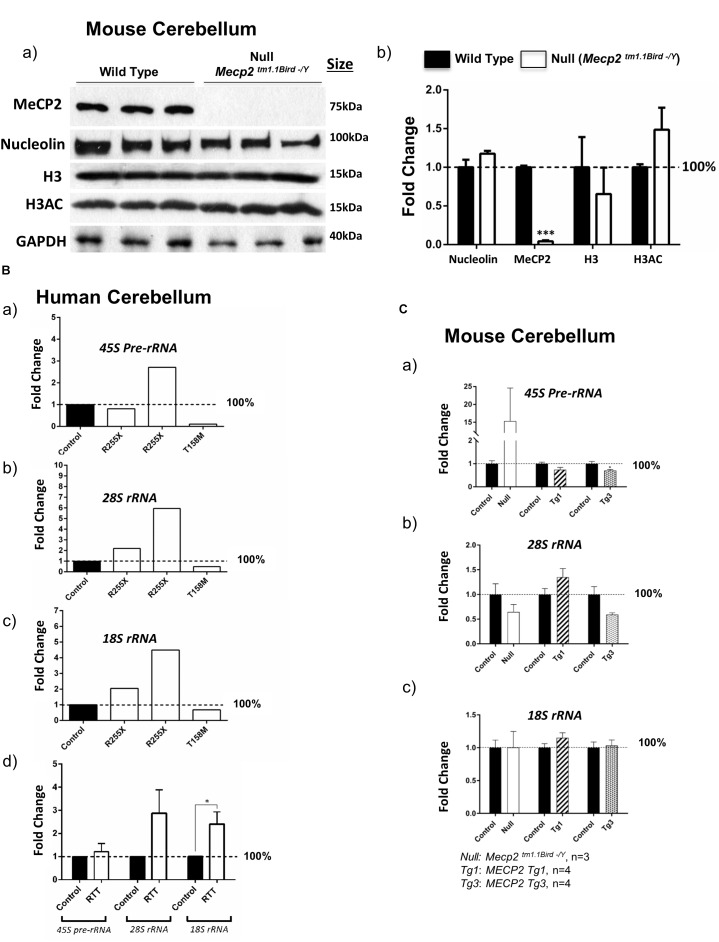
Nucleolin, MeCP2, histone H3, and histone H3 di-acetylation at K9-K14 (H3AC) in the murine cerebellum along with ribosomal RNA transcripts in the human and murine cerebellum. **(A)** Western blot (WB) analysis of the total cell extracts of wild type (WT) and *Mecp2*^*tm*1.1*BirdY*/−^ (null, *N* = 3). As expected, no MeCP2 is detected in the *Mecp2*^*tm*1.1*BirdY*/−^ cerebellum, and nucleolin levels are not changed. No obvious difference in nucleolin, H3, or H3AC is visible between the WT and *Mecp2*^*tm*1.1*BirdY*/−^ null mice **(a)**. Quantification of WB signals is provided in **(b)** confirming no change in the nucleolin, H3, and H3AC levels between the wild-type and null mice. *N* = 3 ± SEM for the WB for WT and null mice. Statistical significance was determined by paired *t*-test, with ^∗∗∗^p < 0.001. **(B)** Transcript levels of *45S* precursor ribosomal RNA (*rRNA*) **(a)**, mature *28SrRNA* **(b)**, and *18SrRNA* **(c)** are shown for the human cerebellum. Human controls are NIH Neurobiobank #5646 and #5446, and RTT patients are in the order of NIH Neurobiobank #4516 and #4882, followed by T158M patient. Means of technical replicates are presented for individual patients. Combined patient data is shown in **(d)** for each rRNA transcript, with N = 2 for controls and *N* = 3 ± SEM for patients. **(C)** Transcript levels of *45S* precursor ribosomal RNA (*rRNA*) **(a)**, mature *28SrRNA* **(b)**, and *18SrRNA* **(c)** are shown for the cerebellum of *Mecp2*^*tm*1.1*BirdY*/−^ (null, *N* = 3), *Tg1* (*MECP2-Tg1*, *N* = 4), and *Tg3* (*MECP2-Tg3*, *N* = 4) mice compared to control cerebellum from WT controls. *N* = 3–4 ± SEM. Statistical significance for Bd and C was determined by Welch’s *t*-test, with ^∗^*p* < 0.05.

Next, we tested whether altered nucleolin levels in the human T158M (higher levels) and R255X (lower levels) is a phenotype that can be detected in one of the most-studied murine RTT mouse models (*Mecp2*^*tm*1.1*Bird/Y*^). In this transgenic mouse, our IHC studies showed no nucleoli structure alteration by nucleolin staining in the cerebellum (Figure 2). The specificity of these detected signals was confirmed by absence of any signal in primary antibody omission control samples (Supplementary Figures S2A–D). In agreement with the absence of nucleoli alteration in murine RTT brain, no change in the nucleolin was detected in *Mecp2*^*tm*1.1*Birdy*/−^ (homozygous, *n* = 3) cerebellum compared to WT (Figure 4A). Accordingly, analysis of H3 and H3AC proteins showed no difference between WT and homozygous *Mecp2*^*tm*1.1*Birdy*/−^ mice (Figure 4B).

### Detection of Ribosomal RNA Transcripts in Human RTT Brain

It has been reported that *Mecp2* deficiency/knockdown in embryonic murine neurons alters the *28S* and *18S rRNA* transcript levels and causes compromised nucleolar structures that are visible during development (Singleton et al., 2011; Gabel et al., 2015). It is also known that nucleolin plays key roles in the transcription of the *45S pre-rRNA* and its processing into mature *rRNAs* (Durut and Saez-Vasquez, 2015). To study whether *rRNA* transcripts are impacted in RTT cerebellum, we analyzed *45S pre-rRNA*, *28S*, and *18S rRNA* transcript levels in these three RTT patients compared to age-matched control cerebellum tissues. While a similar pattern was not observed in these three patients, the two R255X patients (17 and 20 years old) showed a trend of increased *rRNA* transcripts, but T158M cerebellum showed a trend for decreased *rRNA* transcripts (Figure 4B). Regardless, combination of the results from all three patients suggested a trend of increased *rRNA* transcripts, which was significant in case of *18SrRNA* (Figure 4Bd). These results suggest that in RTT brain possible deregulated *rRNA* synthesis might be mutation-dependent, implicating *rRNA* synthesis as a possible contributing mechanism in impaired protein translation that warrants further investigations.

In order to see if ribosomal *RNA* transcripts are affected by absence of MeCP2, we studied *rRNA* transcripts in the cerebellum of 6 weeks null *Mecp2* mice (*Mecp2*^*tm*1.1*Birdy*/−^ homozygous, *n* = 3). While we observed an induction of the *45S pre-rRNA* by 15-fold, no significant difference in processed *28S* and *18S rRNA* transcripts compared to WT mice was detected. Further studies in transgenic mice with overexpressed levels of MeCP2 with either a twofold (*MECP2 Tg1: Tg1*) or a threefold (*MECP2 Tg3: Tg3*) increase in MeCP2 levels, only showed decreased levels of *28S rRNA* in *Tg3* cerebellum, but not *Tg1* mice (*n* = 4). These results suggest that a possible regulation of *rRNA* genes by MeCP2 might be complex and may depend on the type of *MECP2* mutation. It is also possible that MeCP2 represses *rRNA* genes with a trend of *rRNA* induction in *Mecp2*-deficient mice and/or RTT patients and decreased level(s) where MeCP2 is overexpressed (when an alteration is seen in *Tg3* mice) (Figures 4B,C). Such a role for MeCP2 and other MBDs was previously suggested in non-neuronal murine cells (Goshen et al., 2004).

**FIGURE 5:**
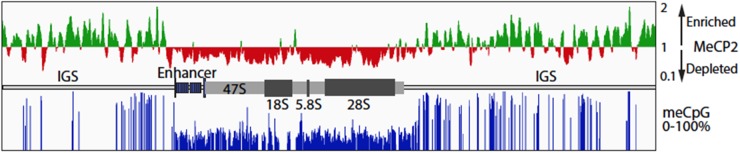
MeCP2 binding at the ribosomal *RNA* gene locus in the murine forebrain. Public data for MeCP2 ChIP-Seq (GSM1464563, GSM1464564) and WGBS (GSM1173783) from 6-week-old mouse forebrain was realigned to the *in silica* mouse genome to which a single copy of the mouse *rRNA* (GenBank BK000964v3) had been added. The upper panel shows the distribution of MeCP2 ChIPped DNA sequence coverage as compared to input DNA sequence coverage. On the vertical scale, regions with a score >1 indicates sequence enrichment and <1 sequence depletion relative the input DNA sequence coverage. MeCP2 is strongly depleted from the enhancer and *47S* transcribed region. The lower panel shows the percent methylation at CpG motifs across the whole *rRNA* gene repeat unit. A diagrammatic representation of the *rRNA* gene organization is shown between the upper and lower panels. IGS, Intergenic Spacer; 47S, the region transcribed into the 47S primary *rRNA* precursor; *18*, *5.8*, and *28S*; the regions encoding the mature *rRNAs*.

### MeCP2 Binding to *rRNA* Genes Follows the meCpG Modification Levels

A significant fraction of the ∼200 *rRNA* mouse and human genes exist in an inactive, highly meCpG modified, and heterochromatin state. Increased *rRNA* gene methylation has been shown to repress *rRNA* transcription (McStay and Grummt, 2008), but unexpectedly, loss of methylation also leads to a repression of transcription that is associated with a failure in *rRNA* processing (Gagnon-Kugler et al., 2009). MeCP2 was suggested to localize to peri-nucleolar areas of the nucleus in the brain (Payen et al., 1998). These condensed chromatin areas include the silent, heterochromatic *rRNA* genes. To examine if MeCP2 binding is indeed present at the *rRNA gene* loci in comparison with meC sites, we performed data mining of published whole genome bisulphite sequencing and MeCP2 ChIP-seq data for 6-week-old mouse frontal cortex samples (Lister et al., 2013; Gabel et al., 2015). Realignment of the ChIP-seq data and its normalization to input sequence coverage revealed MeCP2 enrichment across the Intergenic Spacer (IGS) (Figure 5). By contrast, MeCP2 was depleted from the active gene regions, including the upstream enhancer repeats and the *47S* gene body. CpG methylation of the *rRNA* gene repeat followed a similar pattern, with most sites being fully or nearly fully methylated in the IGS, but on average only 30–40% methylated throughout the active gene regions. No cytidine methylation in the context of non-CpG methylation was detected. Since it is expected that a significant fraction of *rRNA* genes will be heterochromatic, methylation of the active gene regions is consistent with the existence of the silent gene fraction. However, the near 100% methylation observed in the IGS suggests that this region is strongly repressed in all *rRNA* genes regardless of their activity status. Thus, MeCP2 binding followed the overall level of CG methylation, probably being present within the IGS of most *rRNA* genes, but not the gene bodies of the active genes.

**FIGURE 6:**
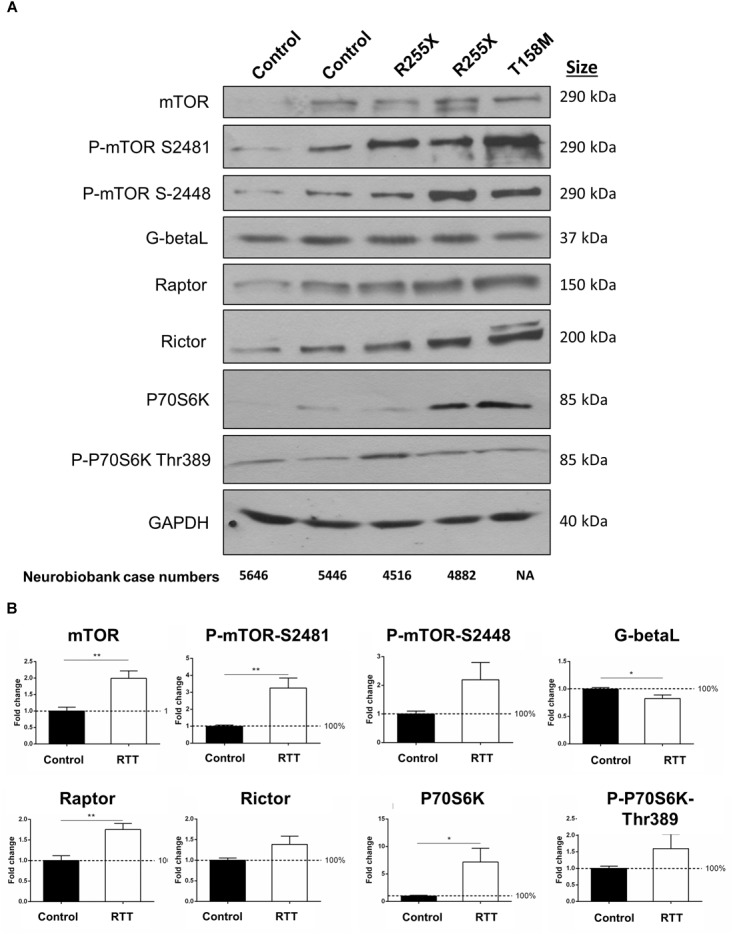
Total and phosphorylated mTOR and P70S6K in the cerebellum of RTT patients. **(A)** Representative Western blots (WB) form total cell extract of human control and RTT cerebellum with indicated antibodies (mTOR, phosphorylated mTOR at Serine 2481 or 2448, G-Beta-L as the common component of mTOR complexes, Raptor as part of mTORC1, and Rictor as part of mTORC2), P70S6K (and its phosphorylated form Thr389) and GAPDH. Molecular weight of each protein is indicated. NIH case numbers are indicated below the blots (NA, not applicable). **(B)** Quantification is reported from two to four WB repeats and average quantification of the corresponding blots from **A** and Supplementary Figure S4. Error bars represent standard error of the mean (SEM), *N* = 3 ± SEM for controls, *N* = 4 ± SEM for RTT patients. Statistical significance was determined by Welch’s *t*-tests with ^∗^*p* < 0.05, or ^∗∗^*p* < 0.01.

### The mTOR–P70S6K Pathway and mTORC1 and mTORC2 Complexes Are Interrupted in RTT Brain

The mTOR–P70S6K pathway is a well-established signaling pathway upstream of protein synthesis. In order to study the protein components of mTOR–P70S6K signaling in human RTT brain, total cell extracts were isolated from the cerebella of controls and RTT patients (two different R255X; a 20-year-old and a 17-year-old patient), one T158M (a 13-year-old patient), and one G451T [a 19-year-old patient with a rare mutation in the MeCP2 C-terminal domain (CTD)]. In all four RTT cerebellum tissues, mTOR levels were about twofold higher than in the controls (Figures 6A,B and Supplementary Figure S4) (^∗∗^*p* < 0.01). Accordingly, the phosphorylated mTOR at both Serine 2448 (mTORC1) and Serine 2481 (mTORC2) were elevated in these RTT patients compared to the controls (^∗∗^*p* < 0.01 for S2481) (Figure 6B). While increased phosphorylation of mTORC2 complex (S2481) was consistent among these four patients, elevated mTORC1 phosphorylation (S2448) was present in R255X and T158M patients (Figure 6A), but absent in G451T mutation (Supplementary Figure S4). This could hint toward differential involvement of MeCP2 protein domains (MBD, TRD, or CTD) in mTORC1 phosphorylation. Despite the activation of mTORC1 and/or mTORC2 complexes (indicated by increased S2448 and S2481 phosphorylation, respectively), the levels of a common protein component of both mTORC1 and mTORC2 complexes, namely, G-Beta-L protein, was slightly, but significantly reduced (^∗^*p* < 0.05) in the RTT cerebellum (Figures 6A,B), suggesting potentially compromised mTORC1/2 functional complexes. Also, the levels of complex-specific protein components of mTORC1 and mTORC2 (Raptor and Rictor, respectively) were slightly (and significantly in the case of Raptor, ^∗∗^p < 0.01) elevated in RTT patients (Figure 6B). While these data indicated that in in these human RTT cerebellums, mTOR protein and its two associated mTORC1 and mTORC2 complexes were elevated, further studies were required to determine if this impacted P70S6K signaling. WB experiments showed that following elevated mTOR, there was also a significant increase of about 7-fold in the protein levels of P70S6K in RTT cerebellum compared to control tissues (^∗^*p* < 0.05) (Figure 6B). Accordingly, phosphorylation of P70S6K at Thr 389 was deregulated in RTT patients (Figures 6A,B), with a drastic decrease in the G451T patient (Supplementary Figure S4). These data collectively may hint toward a deregulated mTORC–P70S6K cell-signaling pathway, in parallel to elevated P70S6K.

**FIGURE 7:**
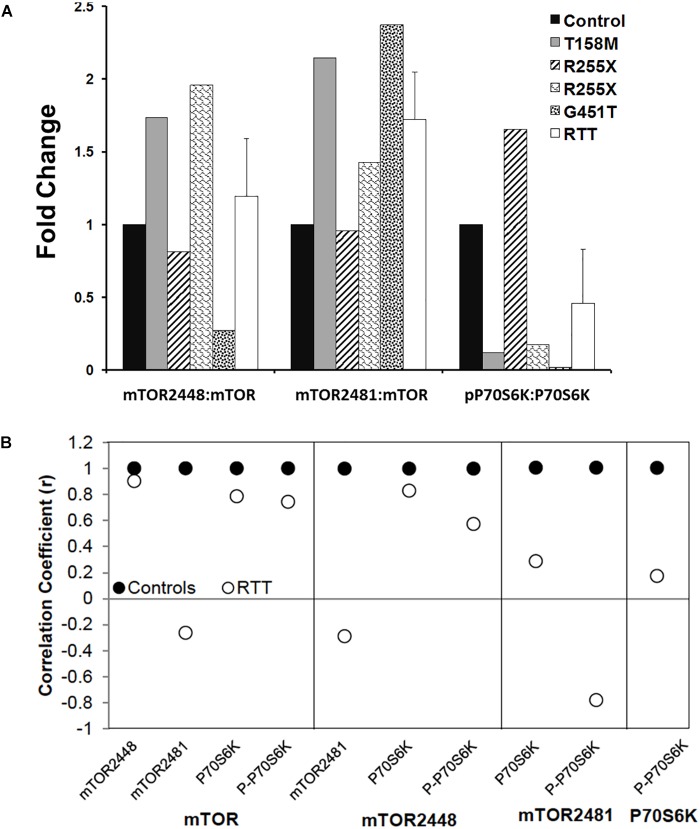
Ratios and correlations of phosphorylated to total protein in Rett syndrome. **(A)** The ratio of individual phosphorylated proteins to the total proteins for indicated molecules is shown. *N* = 3 ± SEM for controls, *N* = 4 ± SEM for RTT patients. **(B)** Correlation coefficients for phosphorylated proteins to total proteins for indicated molecules are shown.

**FIGURE 8:**
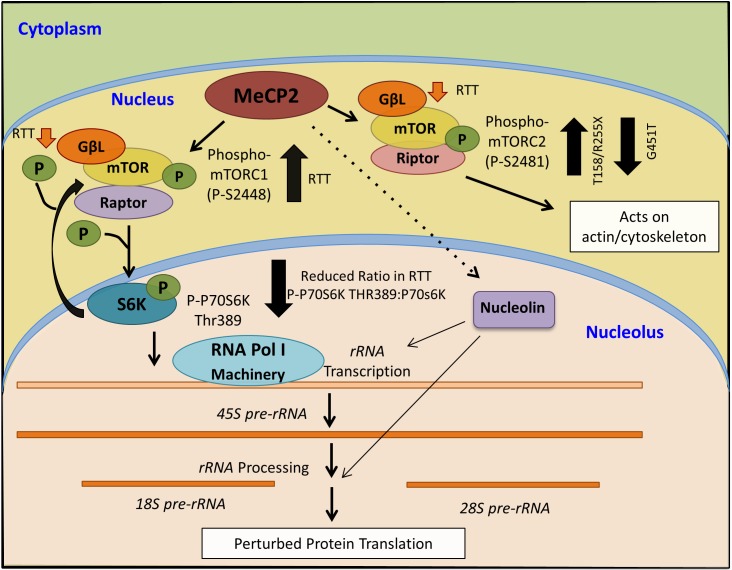
Schematics of a proposed model for MeCP2 involvement in mTOR-P70S6K-nucleolin-*rRNA* synthesis, and its deregulation in Rett syndrome.

Next, we analyzed the ratio of phosphorylated mTORC1 (2448) or mTORC2 (2481) to mTOR (total protein), and phosphorylated P70S6K (Thr389) to P70S6K (total protein). While individual RTT patients did not show similar patterns of the phosphorylated versus non-phosphorylated molecules, a trend of elevated P-mTORC1: mTOR (S2448), P-mTORC2: mTOR (S2481), but reduced P-P70S6K (Thr389): P70S6K was observed compared to the controls (Figure 7A). This prompted us to study the correlation of mTOR, phosphorylated mTORC1 (2448), phosphorylated mTORC2 (S2481), and P70S6K to different components of this pathway, specially phosphorylated P70S6K (Thr389) (Figure 7B). Pearson’s correlation analysis (*r*) showed that while mTOR was similarly correlated with mTORC1 and P-P70S6K-389 in control and RTT brain, mTOR and mTORC2 (S2481) were negatively correlated in RTT patients (Figure 7B). As mTORC2 controls cellular cytoskeleton, this could partly explain the smaller brain size that is a general characteristic of RTT brain. Additionally, mTORC1 (2448) correlation with P-P70S6K was in the moderate range in RTT brain that was lower compared to controls. This is important, as active mTORC1 is the responsible protein directly upstream of phosphorylated P70S6K (Thr389) in this signaling pathway. Notably, similar correlation analysis in RTT patients indicated a weak correlation between P70S6K and its phosphorylated form (P-P70S6K-Thr389). This highlights that in RTT brain, phosphorylation of P70S6K at Thr389, which is required for proper cell signaling toward *rRNA* synthesis and ribosomal biogenesis might be compromised. Thus, impaired mTORC1–P70S6K may be associated with compromised protein synthesis in RTT, a process that is directly downstream of this cellular pathway.

## Discussion

Four common MeCP2 mutations make up >28% of all RTT cases. These include T158M (8.7%) and R168M (7.35%) in the DBD, as well as R255X (6.35%) and R270X (5.8%) in the TRD. We analyzed post-mortem cerebellar tissues from one post-mortem RTT with T158M mutation and two different R255X RTT patients to determine the impact of these common *MECP2* mutations on pathways that impinge on ribosome biogenesis. We provide evidence that nucleolin; a regulator of *rRNA* transcription and processing might be a potential MeCP2 target. In T158M RTT cerebellum, nucleolin levels were changed, along with its nucleolar localization, in association with highly increased protein levels in the cytoplasmic and nuclear fractions of cerebellum. Nucleolin is an RNA-binding protein (Ghisolfi-Nieto et al., 1996) and its abnormal sub-cellular localization may cause molecular abnormalities, besides effects on *rRNA* synthesis and/or processing. In terms of *rRNA* transcription, other well-known MeCP2 targets, such as BDNF and IGF-1, also impinge on *rRNA* transcription (Schratt et al., 2004; Donati et al., 2011) and may therefore play deregulatory roles that warrant further studies.

We also observed increased levels of mTOR and deregulation of its two phosphorylated forms, which contribute to mTORC1 and mTORC2 activities in the brain. Increased mTORC1 phosphorylation in RTT patients (T158M and R255X) may also explain increased levels of p70S6K phosphorylation and/or increased *rRNA* levels. Although not all patients showed a positive trend of *rRNA* increase, it is possible that there are regulatory mechanisms in place that are disturbed, as an opposite change in nucleolin levels (transcripts and protein) is also observed between RTT patients with T158M and R255X mutations. These data suggest that deregulation of cell signaling pathways and molecular properties of RTT brain might be MeCP2 mutation-dependent, which could be addressed by future studies. Our data collectively suggest that in human RTT brain, ribosomal RNA transcripts and/or mTOR–P70S6K may be elevated, pointing toward a potential over-activation of this fundamental process, exhausting cellular resources that are essential for other cellular functions. This may include components of the protein translation machinery, neuronal plasticity, synapse formation, and other critical functions that are compromised in RTT brain and neurons.

While a role for mTOR in autism spectrum disorders is suggested (Onore et al., 2017), to our knowledge, our study is the first to implicate mTOR–P70S6K–nucleolin–*rRNA* synthesis in human RTT cerebellum. Although our results bring important insights toward understanding the molecular abnormalities of the RTT brain, further studies are required to determine if our findings can be generalized to other RTT-associated MeCP2 mutations. RTT is a rare disease with hundreds of different mutations that occur within different protein domains. One limitation of our study was access to a large number of human post-mortem brain tissues with the same mutation. While in our studies, RTT patients showed different trend of nucleolin-*rRNA* biogenesis, two different R255X patients exhibited similar trend of molecular characterization. This suggests that RTT-associated molecular abnormalities might depend on the type of genetic mutations and affected protein domains. Regardless, the mTOR-P70S6K signaling pathway appeared to be more similarly affected between these three patients, suggesting the importance of this pathway in RTT cerebellum. Our analysis of a fourth RTT patient with a rare MeCP2 mutation (G451T) in the MeCP2 CTD highlighted that elevated mTOR and phosphorylation of mTORC2 are common among mutations that involve three different MeCP2 protein domains (MBD, TRD, and CTD). A summary of our results and the proposed model of MeCP2 involvement in the mTOR–P70S6K–nucleolin–*rRNA* synthesis is provided in Figure 8.

## Author Contributions

MR and COO designed experiments. COO dissected murine tissues and prepared protein extracts from murine cerebellum, extracted RNA from *Mecp2*^*tm*1.1*Birdy*/−^ and WT mice, prepared human brain tissues, extracted total protein extracts, prepared nuclear and cytoplasmic extracts, conducted human Western blots, and performed IHC and microscopic imaging. SP extracted RNA from human brain and performed RT-PCR from murine brain. AAS performed mouse WBs and prepared the graph for the generated signals, and marker analysis of nuclear–cytoplasmic extracts by WB. DF performed human brain RT-PCR. DF, DK, KS, and MDB quantified WB results, prepared related graphs and table(s), and correlational co-efficient analysis. YS from Dr. Huda Zoghbi’s lab provided RNA samples from WT, *MECP2*-*Tg1*, and *MECP2*-*Tg3* mice. MS-F and TM conducted alignment analysis of MeCP2 binding at *rRNA* genes. MDB provided control human brain tissues for IHC, and dissected T158M human brain. VMS and LA arranged consent, donation, transfer, and usage of T158M post-mortem brain to the Rastegar lab for research. For controls of RNA and protein extractions, and both R255X RTT brain tissues “Human tissue was obtained from University of Maryland Brain and Tissue Bank, which is a Brain and Tissue Repository of the NIH Biobank.” MR wrote the manuscript, assembled final graphs and images prepared by other authors, provided conception and design, and contributed reagents, materials, analysis tools, and research facilities. All authors have read and approved the final version of the manuscript.

## Conflict of Interest Statement

The authors declare that the research was conducted in the absence of any commercial or financial relationships that could be construed as a potential conflict of interest.

## Footnotes

1 https://github.com/mariFelix/deconvoNorm

## Supplementary Material

FIGURE S1 Detection of nucleolin protein in the cerebellum of R255X Rett syndrome (RTT) patients. **(A,B)** Microscopic images of post-mortem human cerebellum for nucleolin (red) and DAPI signals (white) in two female controls **(a–c)**, and primary omission **(d,e)**. **(C,D)** Microscopic images of post-mortem human cerebellum for nucleolin (red) and DAPI signals (white) in two female R255X patients are shown **(a–c)** as well as the primary omission **(d,e)**. In each panel, the NIH Neurobiobank case number is indicated. GCL, granular cell layer; ML, molecular layer; NCL, nucleolin; PCL, Purkinje cell layer. Scale bars represent 20 μm.

FIGURE S2 Primary omission control for murine cerebellum. Primary antibody omission in wild-type male and female **(A,C)** and mutant homozygote **(B)** or heterozygote **(D)** mice.

FIGURE S3 Quantification of nuclear histone H3 and H3 di-acetylation at K9–K14 (H3AC) and cytoplasmic S100 normalized to GAPDH in Rett syndrome cerebellum and controls. **(A,B)** Western blot (WB) quantification of nuclear cell extracts of controls and RTT patients individually and in combination, respectively. The data are shown with the following samples in the order of controls (NIH NeuroBiobank case numbers #5646 and #5446) and Rett syndrome (RTT) patients (R255X: c.763C>T nonsense mutation, 20 and 17 years old, case numbers #4516 and #4882), and T158M cerebellum (brain received as donation by family members with appropriate consent for research). **(C,D)** Same as in **(A,B)**, but for the cytoplasmic extracts for S100. *N* = 2 for controls while individual patient data is shown in **(A,C)**. For **(B,D)**, *N* = 2 for controls and *N* = 3 ± SEM for RTT patients. Statistical significance was determined by two-way ANOVA, with ^∗∗^*p* < 0.01 and ^∗∗∗∗^*p* < 0.0001.

FIGURE S4 The mTOR and P70S6K signaling molecules in Rett syndrome. Representative Western blots (WB) with total cell extract of a human control and a G451T RTT cerebellum with indicated antibodies (mTOR, phosphorylated mTOR at Serine 2481 or 2448, G-Beta-L as the common component of mTOR complexes, Raptor as part of mTORC1, and Rictor as part of mTORC2), P70S6K (and its phosphorylated form Thr389) and GAPDH. The molecular weight of each detected protein is indicated, and the NIH Neurobiobank case numbers are indicated for the control and RTT cerebellum.

TABLE S1 Primary antibodies used for Western blot (WB) or immunohistochemistry (IHC).

TABLE S2 Secondary antibodies used for Western blot (WB) or immunohistochemistry (IHC).

TABLE S3 Brain sample characteristics for rett syndrome (RTT) patients and controls.

## Abbreviations

DBD: DNA-binding domain
EEG: electroencephalogram
GCL: granular cell layer
IGV: Integrative Genomics Viewer
MBP: methyl binding proteins
MDS: *MECP2* duplication syndrome
*MECP2*: methyl CpG-binding protein 2) (human gene)/*Mecp2* (murine gene)/MeCP2 (protein)
ML: molecular layer
mTOR: mechanistic target of rapamycin
PCL: Purkinje cell layer
PFA: paraformaldehyde
PMI: post-mortem delay
qRT-PCR: quantitative real-time PCR
RPM: reads per million
*rRNA*: ribosomal RNA
RTT: Rett syndrome
SEM: standard error of the mean
TRD: transcriptional repression domain
WB: Western blot
WGBS: whole-genome bisulfite sequencing
WT: wild type.
