# Supplementary figures and images for: MECP2 Mutation Interrupts Nucleolin–mTOR–P70S6K Signaling in Rett Syndrome Patients
Source: Front Genet. 2018 Dec 19;9:635. doi: 10.3389/fgene.2018.00635 (PMC6305968; doi:10.3389/fgene.2018.00635)

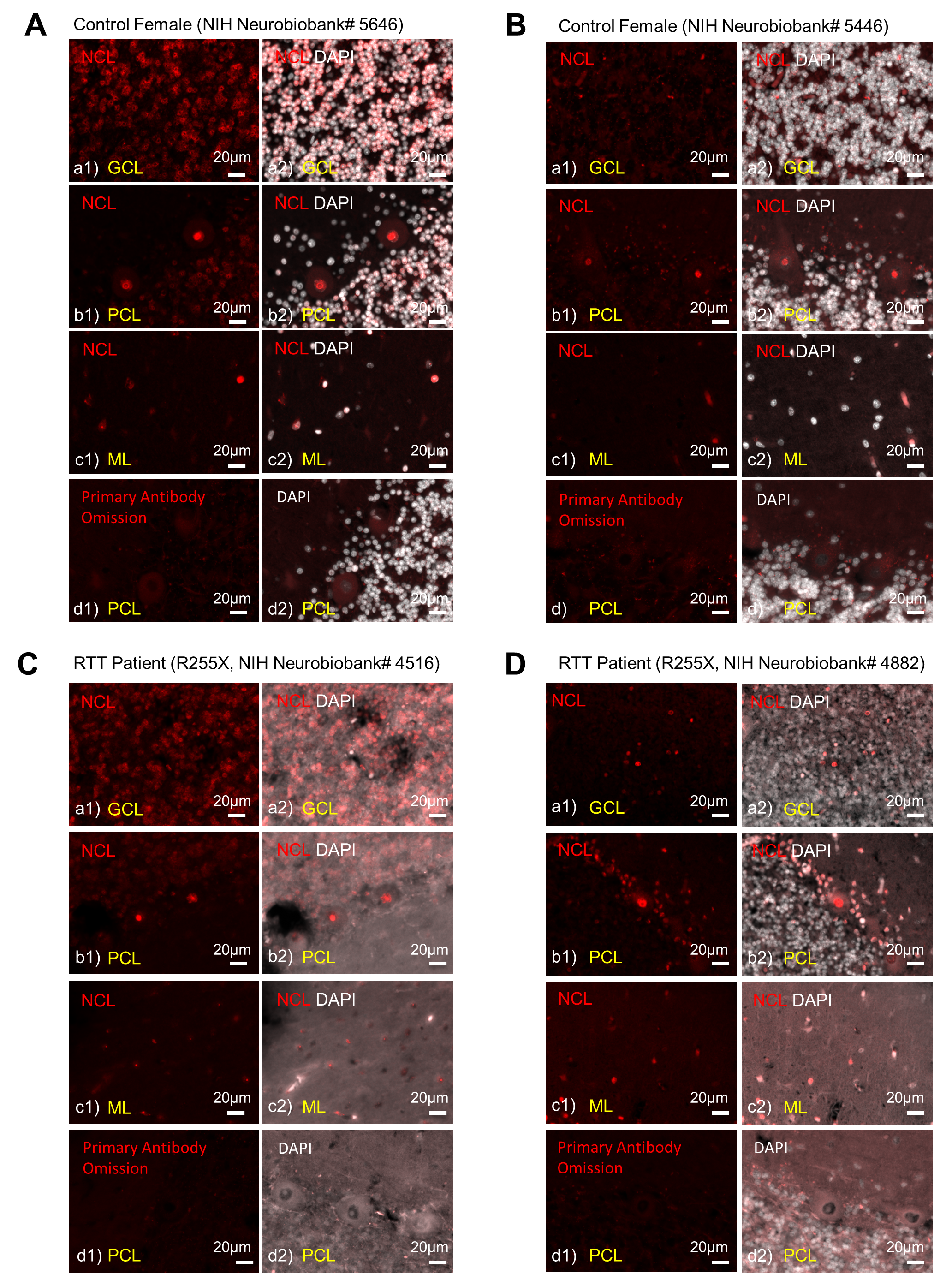

Supplement: FIGURE S1 — Detection of nucleolin protein in the cerebellum of R255X Rett syndrome (RTT) patients. (A,B) Microscopic images of post-mortem human cerebellum for nucleolin (red) and DAPI signals (white) in two female controls (a–c), and primary omission (d,e). (C,D) Microscopic images of post-mortem human cerebellum for nucleolin (red) and DAPI signals (white) in two female R255X patients are shown (a–c) as well as the primary omission (d,e). In each panel, the NIH Neurobiobank case number is indicated. GCL, granular cell layer; ML, molecular layer; NCL, nucleolin; PCL, Purkinje cell layer. Scale bars represent 20 μm. [file Image_1.TIF]

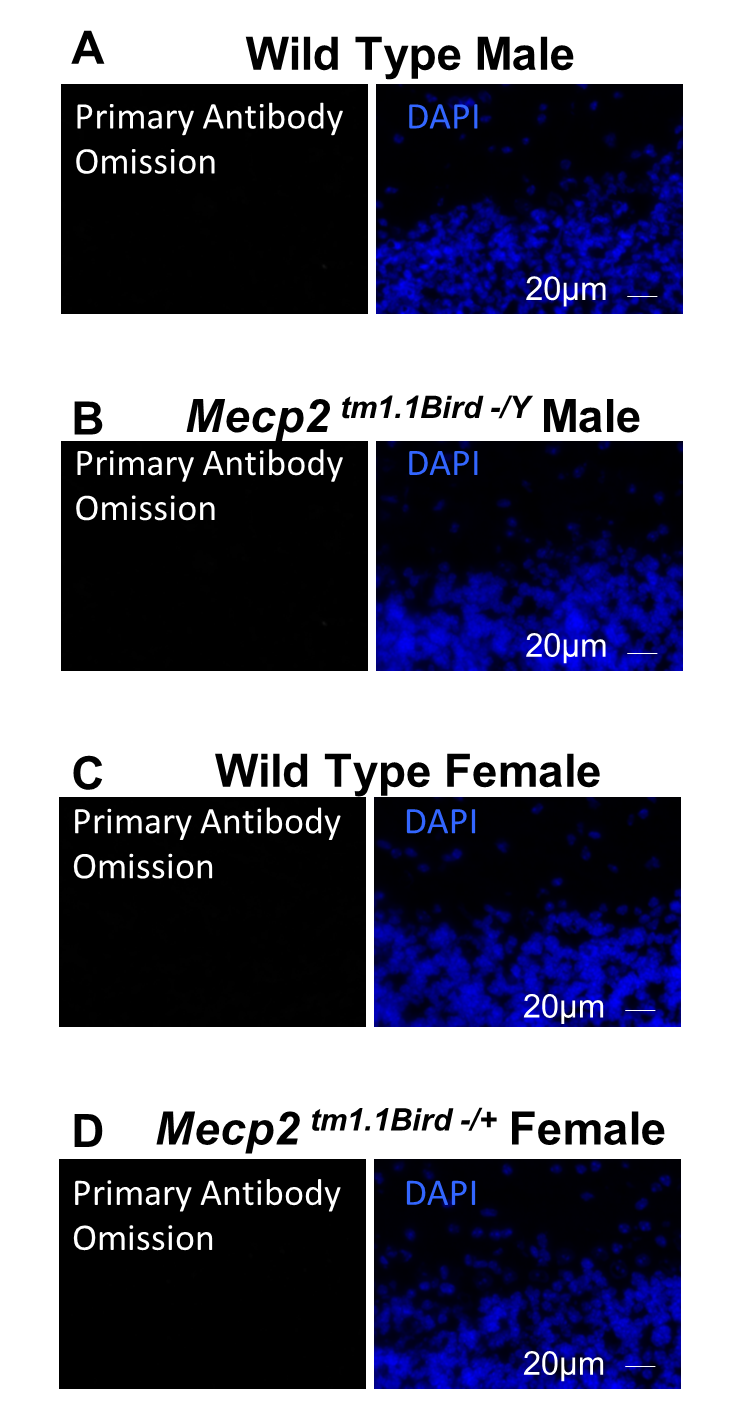

Supplement: FIGURE S2 — Primary omission control for murine cerebellum. Primary antibody omission in wild-type male and female (A,C) and mutant homozygote (B) or heterozygote (D) mice. [file Image_2.TIF]

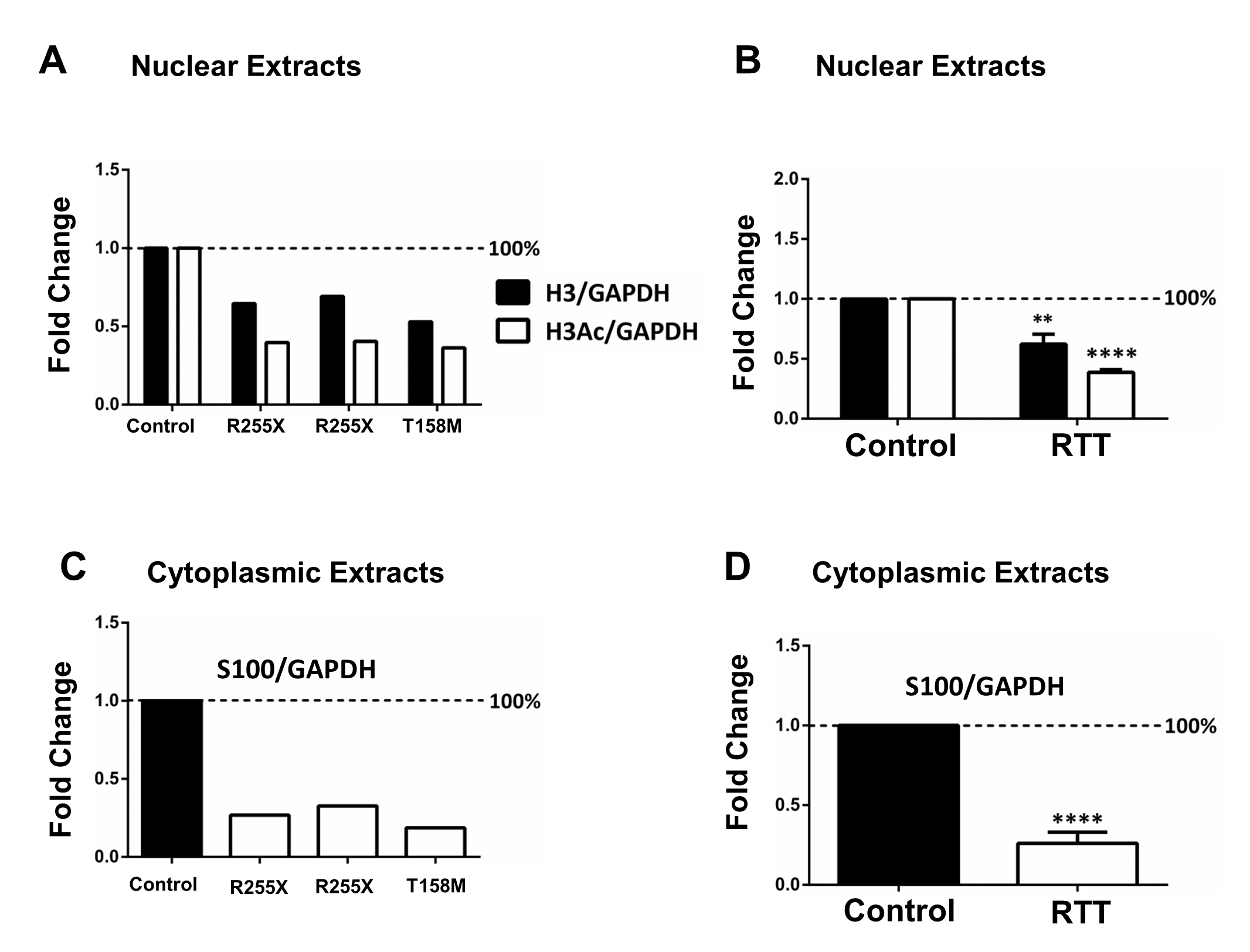

Supplement: FIGURE S3 — Quantification of nuclear histone H3 and H3 di-acetylation at K9–K14 (H3AC) and cytoplasmic S100 normalized to GAPDH in Rett syndrome cerebellum and controls. (A,B) Western blot (WB) quantification of nuclear cell extracts of controls and RTT patients individually and in combination, respectively. The data are shown with the following samples in the order of controls (NIH NeuroBiobank case numbers #5646 and #5446) and Rett syndrome (RTT) patients (R255X: c.763C>T nonsense mutation, 20 and 17 years old, case numbers #4516 and #4882), and T158M cerebellum (brain received as donation by family members with appropriate consent for research). (C,D) Same as in (A,B), but for the cytoplasmic extracts for S100. N = 2 for controls while individual patient data is shown in (A,C). For (B,D), N = 2 for controls and N = 3 ± SEM for RTT patients. Statistical significance was determined by two-way ANOVA, with ∗∗p < 0.01 and ∗∗∗∗p < 0.0001. [file Image_3.TIF]

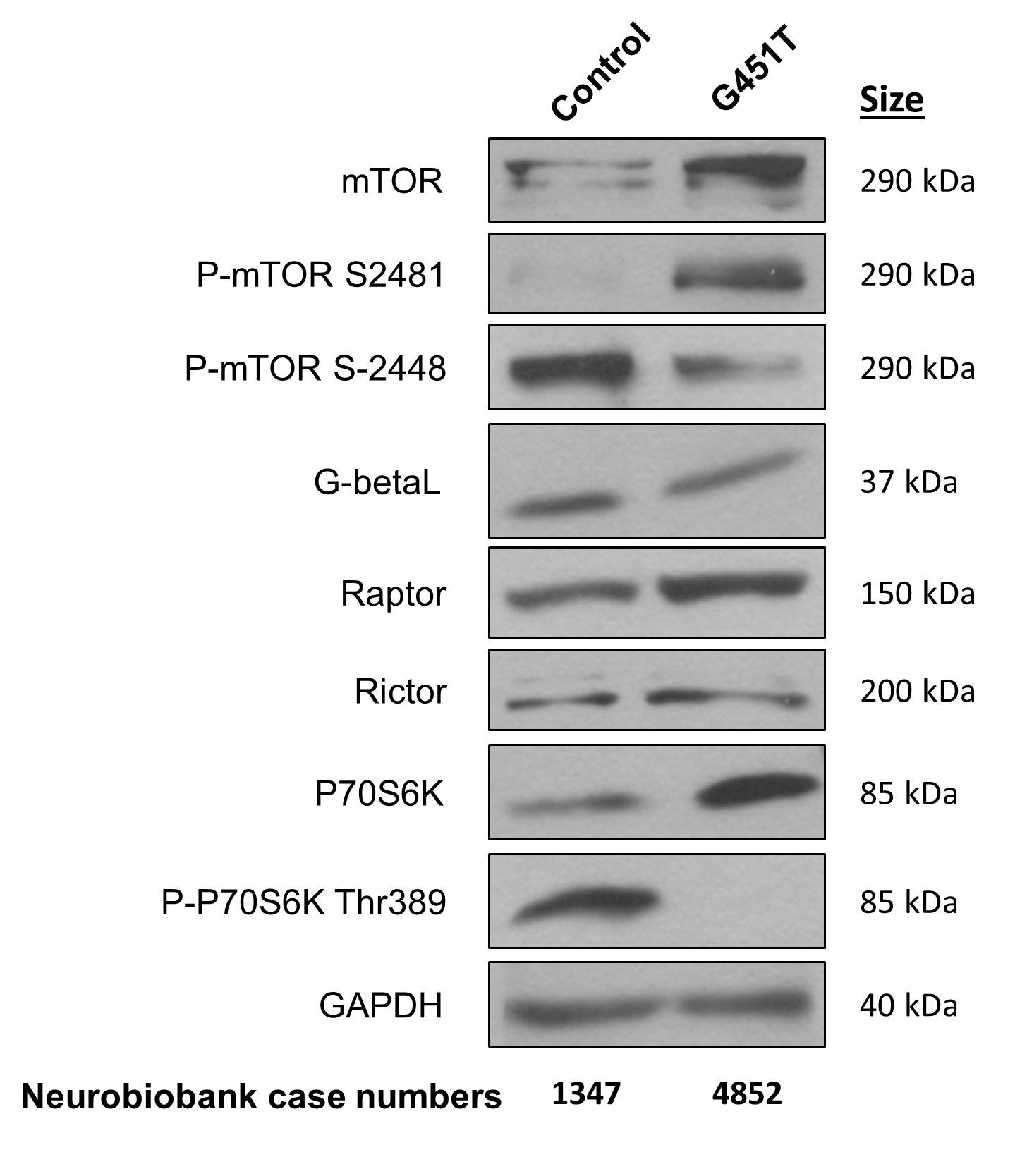

Supplement: FIGURE S4 — The mTOR and P70S6K signaling molecules in Rett syndrome. Representative Western blots (WB) with total cell extract of a human control and a G451T RTT cerebellum with indicated antibodies (mTOR, phosphorylated mTOR at Serine 2481 or 2448, G-Beta-L as the common component of mTOR complexes, Raptor as part of mTORC1, and Rictor as part of mTORC2), P70S6K (and its phosphorylated form Thr389) and GAPDH. The molecular weight of each detected protein is indicated, and the NIH Neurobiobank case numbers are indicated for the control and RTT cerebellum. [file Image_4.TIF]
