# Supplementary material for: MECP2 Mutation Interrupts Nucleolin–mTOR–P70S6K Signaling in Rett Syndrome Patients
Source: Front Genet. 2018 Dec 19;9:635. doi: 10.3389/fgene.2018.00635 (PMC6305968; doi:10.3389/fgene.2018.00635)
Supplement: TABLE S1 — Primary antibodies used for Western blot (WB) or immunohistochemistry (IHC). [file Table_1.pdf]

Supplementary Table 1. Primary antibodies used for western blot (WB) or immunohistochemistry (IHC).

Target	Company	Cat #	Host	Blocking	Dilution
S100 (4C4.9)	Abcam	Ab4066	Mouse	5% skim milk	1:200
Histone H3	Abcam	Ab1791	Rabbit	5% skim milk	1:1000
MeCP2	ThermoFisher	PA5-12234	Rabbit	5% skim milk	1:1000
Histone H3 di-acetyl (K9-K14) (H3 Ac)	Millipore	06-599	Rabbit	5% skim milk	1:2000
Nucleolin	Abcam	Ab22758	Rabbit	WB: 3% skim milk<br>IHC: 10% NGS	WB: 1:1000<br>IHC: 1:500
mTOR	ThermoFisher	PA 1-518	Rabbit	3% skim milk	1:500
P-mTOR-S2481	ThermoFisher	PA5-38374	Rabbit	3% skim milk	1:1000
P-mTOR-S2448	ThermoFisher	44-1125G	Rabbit	3% skim milk	1:1000
GAPDH	Santa Cruz	Sc-25778	Rabbit	3% skim milk	1:5000
mTOR (7C10)	Cell Signalling Technologies	2983	Rabbit	5% BSA	1:1000
P-mTOR-S2481	Cell Signalling Technologies	2974	Rabbit	5% BSA	1:1000
P-mTOR-S2448	Cell Signalling Technologies	5536	Rabbit	5% BSA	1:1000
Raptor (24C12)	Cell Signaling Technology	2280	Rabbit	5% BSA	1:1000
Rictor (53A2)	Cell Signaling Technology	2114	Rabbit	5% BSA	1:1000
G beta L	Cell Signaling Technology	3274	Rabbit	5% BSA	1:1000
P70S6K (49D7)	Cell Signaling Technology	2708	Rabbit	5% BSA	1:1000
P-p70S6K-Thr389	Cell Signaling Technology	9205	Rabbit	5% BSA	1:1000
Beta-actin	Sigma	A228	Mouse	3% skim milk	1:500
α-tubulin-DM1A	Sigma	T9026	Mouse	3% skim milk	1:500
