# Supplementary material for: MECP2 Mutation Interrupts Nucleolin–mTOR–P70S6K Signaling in Rett Syndrome Patients
Source: Front Genet. 2018 Dec 19;9:635. doi: 10.3389/fgene.2018.00635 (PMC6305968; doi:10.3389/fgene.2018.00635)
Supplement: TABLE S2 — Secondary antibodies used for Western blot (WB) or immunohistochemistry (IHC). [file Table_2.pdf]

Supplementary Table 2. Secondary antibodies used for western blot (WB) or immunohistochemistry (IHC).

Target	Company	Cat #	Host	Blocking	Dilution
Goat anti-Rabbit IgG (H+L) Highly Cross-Adsorbed Secondary Antibody, Alexa Fluor 594	ThermoFisher Scientific	A-11037	Goat	IHC: 10% Normal Goat Serum	1:1000
Peroxidase-conjugated AffiniPure $F(ab')_2$ fragment Donkey anti-rabbit IgG (H+L)	Jackson ImmunoResearch Laboratories, Inc.	711-036-152	Donkey	5% BSA or 3% skim milk	1:15000
Peroxidase-conjugated AffiniPure Sheep anti-mouse IgG (H+L)	Jackson ImmunoResearch Laboratories, Inc.	515-035-062	Sheep	5% BSA or 3% skim milk	1:15000
Anti-Rabbit IgG-peroxidase produced in goat, Affinity isolated antibody	Sigma-Aldrich	A6154	Goat	3% skim milk	1:5000
Goat anti-mouse IgG	Jackson ImmunoResearch Laboratories, Inc.	115-035-174	Goat	3% skim milk	1:7500
