# Supplementary material for: MECP2 Mutation Interrupts Nucleolin–mTOR–P70S6K Signaling in Rett Syndrome Patients
Source: Front Genet. 2018 Dec 19;9:635. doi: 10.3389/fgene.2018.00635 (PMC6305968; doi:10.3389/fgene.2018.00635)
Supplement: TABLE S3 — Brain sample characteristics for rett syndrome (RTT) patients and controls. [file Table_3.pdf]

Supplementary Table 3. Brain Sample Characteristics for Rett Syndrome (RTT) patients and controls.

**NIH Neurobaiobank Sample Number #**	***MECP2* Mutation**	**Known Disease**	**Age (Y.D)**	**PMI**	**Storage (Y.D)** (Prior arriving to our lab)	**Cause of Death** (When known)
NA/organ donation	T158M	Rett Syndrome	>12	< 6 h	Less than two weeks	Respiratory failure
NIH # 4516	R255X	Rett Syndrome	20.356	9h	11.360	Natural
NIH # 4882	R255X	Rett Syndrome	17.310	18 h	9.131	Complication of Disorder
NIH # 4852	G451T	Rett Syndrome	19.280	13h	9.357	Seizures
NA/provided by Dr. Marc Del Bigio (co-author)	Control	Unaffected	14	24 h	2 weeks	Hanging
1347	Control	Unaffected	19.76	16h	14.336	Accidental-multiple injuries
5646	Control	Acute reactive airway disease	20.163	23h	2.308	Medical
5446	Control	Reactive airway disease	17.117	18h	4.58	Natural cause

PMI: Post-mortem interval
Y.D: Year.Day
